# Metabolic alkalosis masked presentation of diabetic ketoacidosis: A case report

**DOI:** 10.1002/ccr3.8250

**Published:** 2023-11-27

**Authors:** Thunyatorn Wuttiputhanun, Natavudh Townamchai, Somchai Eiam‐Ong, Kullaya Takkavatakarn

**Affiliations:** ^1^ Division of Nephrology, Department of Medicine King Chulalongkorn Memorial Hospital and Chulalongkorn University Bangkok Thailand

**Keywords:** diabetic ketoacidosis, gastric alkalosis, ketoalkalosis, metabolic alkalosis

## Abstract

Managing mixed acid–base disorders can be diagnostically challenging, particularly when metabolic acidosis and metabolic alkalosis occur simultaneously. When dealing with metabolic alkalosis, a comprehensive approach involves taking a detailed medical history, assessing volume status, and performing urine chloride analysis. Routine calculation of the anion gap is important to identify masked wide anion gap metabolic acidosis. We report a case of a 32‐year‐old female with type 1 diabetes mellitus, presented with intractable vomiting for 2 days with hyperglycemia, hypokalemia, and metabolic alkalosis, along with a wide anion gap. She was diagnosed with “diabetic ketoalkalosis” due to diabetic ketoacidosis combined with vomiting‐induced metabolic alkalosis. She became clinically stable after resuscitation with normal saline, intravenous potassium, and intravenous insulin.

## Graphical abstract

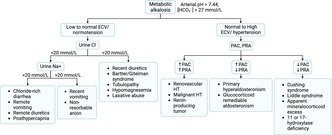

## 1 BACKGROUND

Diabetic ketoacidosis (DKA) is a serious complication of diabetes. The main pathogenesis of DKA is the insufficiency of effective insulin and increased levels of counterregulatory hormones leading to hyperglycemia and ketosis. DKA is diagnosed when patients have hyperglycemia (plasma glucose concentration ≥250 mg/dL), with wide anion gap metabolic acidosis (pH <7.30 and serum bicarbonate ≤18 mmol/L), and the presence of ketonemia or ketonuria. Promptly appropriate treatment with the administration of intravenous fluid and insulin, along with monitoring of an electrolyte disturbance, leads to better outcomes and fewer complications. However, some DKA patients might present with metabolic alkalosis resulting in delayed diagnosis and treatment. The uncommon manifestation of DKA in this patient is known as ketoalkalosis or alkaline ketoacidosis which can occur when acidosis is overridden by coexisting alkalosis.

## 2 CASE REPORT

A 32‐year‐old female with type 1 diabetes mellitus was admitted due to intractable vomiting and fatigue for 2 days. Three days prior to admission, she used methamphetamine and got euphoria. She denied having any food and missed the insulin. One day later, she developed nausea and intractable vomiting more than 10 times. On admission, she was afebrile but lethargic. Her blood pressure was 100/70 mmHg, her pulse rate was 128 beats per minute, and her respiratory rate was 18 per minute without Kussmaul breathing. She had dry lips and a dry tongue. Her abdomen was soft with no tenderness.

Initial laboratory values revealed plasma glucose 313 mg/dL, serum sodium 125 mmol/L, potassium 2.5 mmol/L, chloride 69 mmol/L, bicarbonate 37 mmol/L, anion gap 19 mmol/L, blood urea nitrogen 16 mg/dL, and creatinine 0.5 mg/d. Beta‐hydroxybutyrate was 3.4 mmol/L (normal <0.6 mmol/L). Venous blood gas analysis showed pH 7.502, pCO2 47.7 mmHg, and HCO_3_^−^ 37 mmol/L. Urine analysis showed ketone 2+ and glucose 2+. Her urine sodium was 32 mmol/L, urine potassium 30 mmol/L, and urine chloride <20 mmol/L (Table 1).

**TABLE 1:** Laboratory results.

Parameter	Result	Reference
Serum		
Sodium, mmol/L	125	135–145
Potassium, mmol/L	2.5	3.5–5.0
Chloride, mmol/L	69	98–107
Bicarbonate, mmol/L	37	22–29
Anion gap, mmol/L	19	8–12
Albumin, g/dL	4.6	3.5–5.0
Urea nitrogen, mg/dL	16	7–20
Creatinine, mg/dL	0.5	0.5–1.0
Glucose, mg/dL	312	70–100
Beta‐hydroxybutyrate, mmol/L	3.4	<0.6
Venous blood gas		
pH 7.502, pCO_2_ 47.7 mmHg, HCO_3_^−^ 37.6 mmol/L, pO_2_ 34 mmHg		
Urine		
Specific gravity	1.015	1.003–1.030
pH	7.0	
Sodium, mmol/L	32	
Potassium, mmol/L	30	
Chloride, mmol/L	<20	
Creatinine, mg/dL	63.4	
Urine K/Urine Cr, mmol/g	47.3	

## 3 DIAGNOSTIC ASSESSMENT

The initial investigations demonstrated the presence of hyperglycemia, hyponatremia, hypokalemia, and metabolic alkalosis. Hyponatremia, which corrected sodium was 128.4 mmol/L after considering the effect of hyperglycemia, along with signs of volume depletion, may arise from hypovolemia hyponatremia. However, urine sodium, which is commonly used as an indicator of effective intravascular volume, may not be as reliable in the setting of metabolic alkalosis. The presence of bicarbonaturia from metabolic alkalosis leads to increasing excretion of cations, including sodium and potassium, in urine. Instead, urine chloride is a more suitable marker to use. Low urine chloride, high urine sodium, and signs of hypovolemia in the presence of metabolic alkalosis as seen in this patient, support that metabolic alkalosis is resulted from recent vomiting. The approach to addressing metabolic alkalosis is described in the discussion section.

Additionally, despite the presence of alkalemia, it is recommended to calculate the anion gap to explore the possibility of concurrent mixed‐wide anion gap metabolic acidosis. In our case, the calculated anion gap of 19 mmol/L, with the patient's underlying disease of type 1 diabetes mellitus and hyperglycemia, led the medical team to suspect diabetic ketoacidosis as a potential cause. An elevated serum beta‐hydroxybutyrate level of 3.4 mmol/L confirmed the diagnosis of diabetic ketoacidosis.

## 4 TREATMENT AND OUTCOME

After being diagnosed with diabetes ketoacidosis with alkalemia, she was initially treated with intravenous normal saline 2500 mL in the first 2 h. The insulin drip was started after the potassium was raised to 3.8 mmol/L by potassium intravenous supplement (total of 80 mmol in 4 h). After 18 h, the patient received 4 L of intravenous fluid (normal saline and 5% dextrose saline) and an insulin intravenous drip of 1.5 units/h. The patient's symptoms, including nausea and vomiting, were resolved. Her blood glucose was 131 mg/dL, serum sodium 137 mmol/L, potassium 3.4 mmol/L, chloride 102 mmol/L, bicarbonate 28 mmol/L, anion gap 7 mmol/L, and beta‐hydroxybutyrate 0.7 mmol/L. Regular insulin of 8 units subcutaneously was prescribed and intravenous fluid, and insulin were discontinued 1 h later. After 3 days of admission, the patient was discharged with premixed human insulin (70/30) 14‐0‐10‐unit subcutaneous premeal.

One month after discharge, she was appointed at the outpatient clinic and was clinically stable. She complied with medication and denied any substance use. Fasting plasma glucose was 155 mg/dL, and serum electrolytes became normal. Insulin was adjusted according to self‐monitoring blood glucose and carbohydrate counting.

## 5 DISCUSSION

In our patient, multiple electrolyte disturbances were observed, requiring a careful approach to each problem. Her main electrolyte disorders, which are metabolic alkalosis with masked wide anion gap metabolic acidosis and hypokalemia, and their pathophysiology are discussed as follows.

### 5.1 Metabolic alkalosis

**FIGURE 1:**
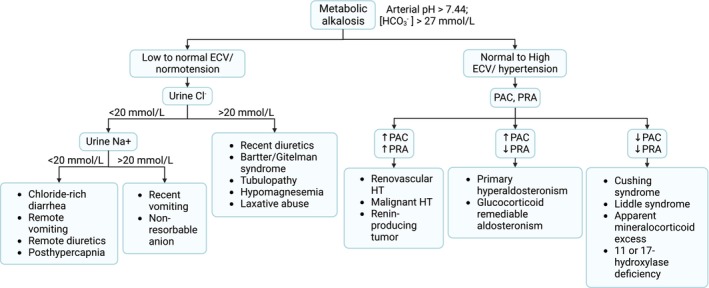
Algorithm for an approach to metabolic alkalosis. ECF, extracellular fluid; HT, hypertension; PAC, plasma aldosterone concentration; PRA, plasma renin activity. Created by BioRender.com.

To determine the causes of metabolic alkalosis, a comprehensive evaluation including history, volume status, kidney function, and urine electrolytes should be performed. Since bicarbonaturia can induce high urine sodium (Na^+^) despite volume depletion in metabolic alkalosis, urine Cl^−^ is used instead of urine Na^+^ to represent volume status. Low urine chloride (Cl^−^) indicates low effective circulating volume (ECV), while high urine Cl^−^ suggests a volume‐expanded state or defect in renal tubular reabsorption. Based on urine Cl^−^, causes of metabolic alkalosis can be divided into chloride‐responsive (urine Cl^−^ <20 mmol/L) and chloride‐resistant (urine Cl^−^ ≥20 mmol/L)^1^ as shown in Figure 1.

In this case, the patient had a history of intractable vomiting and clinical volume depletion. Urine electrolytes revealed low urine Cl^−^, which is consistent with hypovolemia status from vomiting. High urine Na^+^ and urine pH > 6.5 are indicative of bicarbonaturia, a response expected in the context of alkalemia.

The pathogenesis of metabolic alkalosis consists of two distinct factors: generation and maintenance. The generation of metabolic alkalosis is mainly due to excess bicarbonate (HCO_3_^−^) accumulation from endogenous or exogenous sources. The loss of acid through the gastrointestinal tract or kidney is directly correlated with the generation of HCO_3_^−^. Normally, the kidney has a great capacity to excrete excess HCO_3_^−^ into the urine. The presence of the maintenance factors which are the mechanisms that impair kidney HCO_3_^−^ excretion results in persistent metabolic alkalosis. Table 2 summarizes the generation and maintenance factors of metabolic alkalosis.

**TABLE 2:** Summarizes the generating and maintenance factors of metabolic alkalosis (adapted from^2^, ^3^).

Generating factors	Maintenance factors
“Excessive HCO_3_^−^ loads” *Exogenous HCO*_*3*_^*−*^ 1.1 Absolute: loads of NaHCO_3_ or alkalinizing Na salts e.g., lactate, acetate, citrate 1.2 Relative: alkaline loads in kidney failure *Endogenous HCO*_*3*_^*−*^ 2.1 Gastrointestinal tract (via HCl loss): vomiting, nasogastric suction, chloride‐rich diarrhea 2.2 Kidney: (HCO_3_^−^ generation via enhanced H^+^ secretion) 2.2.1 Hyperaldosteronism 2.2.2 Increased distal Na^+^ delivery to collecting duct Diuretics Tubulopathy Na^+^ salt of nonabsorbable anion	“Impaired HCO_3_^−^ removal” Decreased glomerular filtration rate Hyperaldosteronism Hypochloremia Hypokalemia

*Note*: Vomiting or gastric alkalosis contributes to both generation and maintenance factors of metabolic alkalosis.

### 5.2 Generation

Gastric parietal cells produce gastric acid by generating intracellular H^+^ and HCO_3_^−^ from H_2_O and CO_2_ using the carbonic anhydrase II enzyme. H^+^ is secreted along with Cl^−^ into the gastric lumen, and HCO_3_^−^ is absorbed into the blood via basolateral Cl^−^/HCO_3_^−^ anion exchange protein 2 (alkaline tide). Normal gastric acid production, however, does not result in metabolic alkalosis. While gastric H^+^ enters the small bowel, it will be neutralized by HCO_3_^−^ secreted primarily by the pancreas. The secretion of pancreatic HCO_3_^−^ into the intestinal lumen also adds the same amount of H^+^ to the body fluids as the amount of HCO_3_^−^ gained from gastric production thereby maintaining the acid–base balance.^4^

**FIGURE 2:**
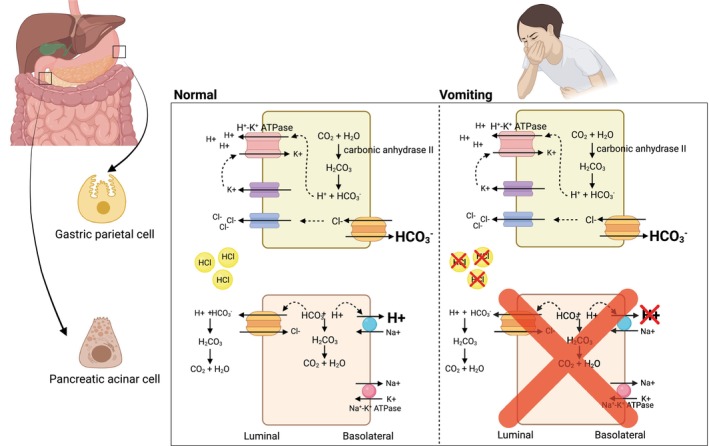
Acid–base regulation in the gastrointestinal tract during normal and vomiting stages. Normal gastric secretion by parietal cells: H^+^ and Cl^−^ are secreted into the gastric lumen while HCO_3_^−^ is absorbed into the blood. When HCl from the stomach enters the small intestine, pancreatic cells secrete HCO_3_^−^ to neutralize the acid and add H^+^ back into the circulation, thereby maintaining acid–base balance (Left). In case of vomiting, HCl is eliminated from the body, preventing pancreatic HCO_3_^−^ secretion, resulting in a net increase in HCO_3_^−^ and the development of metabolic alkalosis (Right). Created by BioRender.com.

Vomiting or nasogastric tube suction removes the gastric HCl from the body and prevents it from reaching to small bowel resulting in the absence of pancreatic HCO_3_^−^ secretion into the intestinal lumen. Without H^+^ gained during pancreas HCO_3_^−^ secretion, HCO_3_^−^ added from gastric production leads to HCO_3_^−^ excess and generates metabolic alkalosis (Figure 2).

### 5.3 Maintenance

**FIGURE 3:**
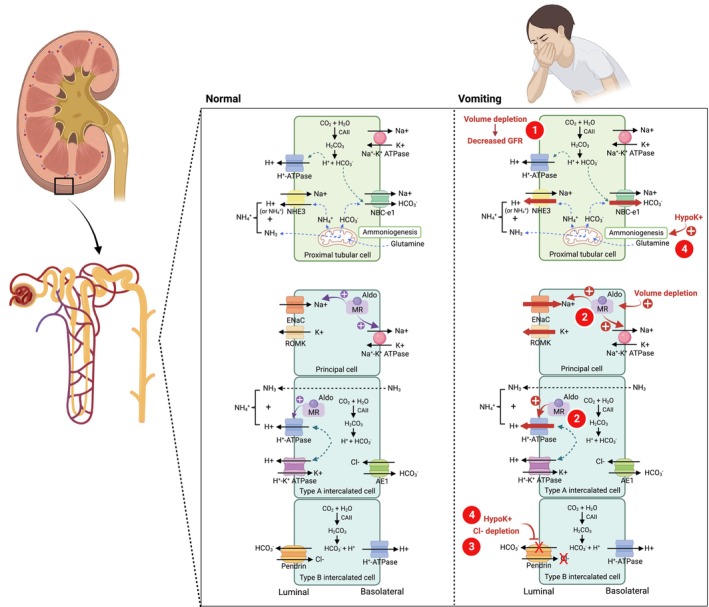
Mechanisms of metabolic alkalosis maintenance in vomiting. (1) Volume contraction decreases GFR and HCO_3_^−^ filtration. (2) Hyperaldosteronism stimulated by volume contraction increases acid secretion at the distal tubule and collecting duct. (3) Hypochloremia impairs HCO_3_^−^ secretion from pendrin (Cl^−^/HCO_3_^−^ exchanger) at β‐intercalated cell. (4) Hypokalemia leading to intracellular acidosis and increasing ammoniogenesis at the proximal tubule, enhances ammonium (NH_4_^+^) secretion and gains the new HCO_3_^−^. Hypokalemia also downregulates pendrin and decreases HCO_3_^−^ secretion at β‐intercalated cells. Aldo, aldosterone; AE1, anion exchanger 1; CAII, carbonic anhydrase II; ENaC, epithelial sodium channel; MR, mineralocorticoid receptor; NBCe1, electrogenic sodium bicarbonate cotransporter1; NHE3, sodium hydrogen exchanger 3; ROMK, renal outer medullary potassium channel. Created by BioRender.com.

Vomiting maintains metabolic alkalosis through four factors (Figure 3): (1) decreased glomerular filtration rate, (2) hyperaldosteronism state (3) hypochloremia, and (4) hypokalemia.^1^

1. *Decreased glomerular filtration rate*: Volume loss after vomiting results in decreased kidney perfusion and glomerular filtration rate (GFR). This state reduces the amount of filtered HCO_3_^−^ and impairs excess HCO_3_^−^ removal from intravascular part.
2. *Hyperaldosteronism*: Volume contraction activates the renin‐angiotensin‐aldosterone system (RAAS) which secondary hyperaldosteronism stimulates Na^+^ reabsorption exchanged with potassium (K^+^) and H^+^ secretion in distal tubule resulting in hypokalemia and metabolic alkalosis.
3. *Hypochloremia*: Chloride loss from vomiting or chloride‐rich diarrhea results in hypochloremia which impair HCO_3_^−^ excretion via pendrin in β‐intercalated cell.
4. *Hypokalemia*: Hypokalemia contributes to metabolic alkalosis via the following mechanisms: (1) stimulate ammoniogenesis in proximal tubule due to intracellular acidosis state, (2) increasing proximal tubular reabsorption of filtered HCO_3_^−^, (3) enhancing H^+^ secretion via H^+^‐ATPase and AE1 activation in α‐intercalated cells, and (4) decreasing HCO_3_^−^ secretion by downregulating the expression of pendrin in β‐intercalated cells.^4^

During **active or recent vomiting**, bicarbonaturia results in high urine Na^+^ and high urine pH. However, in the event of **inactive or remote vomiting** which vomiting is resolved but volume status is still depleted, HCO_3_^−^ excretion is further worsening due to GFR reduction, hypochloremia, and hypokalemia. Low urine pH (pH 6 or lower) occurs despite the presence of metabolic alkalosis, called “paradoxical aciduria.” Low urine Na^+^ and low urine K^+^ are also noted due to the hyperaldosteronism effect and absence of bicarbonaturia.^4^

### 5.4 Hypokalemia

The gastric fluid typically contains 5–10 mmol/L of K^+^.^5^ To create hypokalemia, the patient would need to lose gastric content of approximately 20–40 L, which is unachievable. Actually, the main mechanism of hypokalemia in vomiting is through renal K^+^ loss. This is supported by high urine K^+^ and urine K/urine creatinine as shown in her laboratory result. As discussed above, vomiting leads to bicarbonaturia which increases the luminal electrochemical gradient and enhances tubular K^+^ excretion. Volume depletion after vomiting activates RAAS resulting in secondary hyperaldosteronism which stimulates H^+^ and K^+^ excretion in the cortical collecting duct. In addition, DKA also contributes to tubular K^+^ loss via osmotic diuresis in hyperglycemia and the anionic property of ketoacidosis.

### 5.5 Masked wide anion gap metabolic acidosis

This insulin‐dependent diabetic patient with a history of insulin omission presented with high blood sugar levels and elevated ketonemia, making DKA suspicious. However, due to the absence of metabolic acidosis as a diagnostic criterion, physicians may delay or be hesitant to make a DKA diagnosis.

An anion gap is an important tool in this situation. A high anion gap represents an increase in unmeasured anions or a decrease in unmeasured cations. Theoretically, severe metabolic alkalosis may be associated with a small increment of anion gap by the effect of alkaline pH on the electrical charge of albumin.^6^ However, metabolic alkalosis alone is insufficient to cause substantial changes in the serum anion gap. In this case, the serum anion gap was significantly increased suggesting the presence of an unmeasured anion masked by metabolic alkalosis, most likely from ketone based on the patient's medical history. Therefore, the calculation of the anion gap is recommended in all patients with an acid–base disorder in order to enhance the understanding of patient conditions.

Diabetic alkalosis, the setting of alkalemia combined with diabetic ketoacidosis, was reported in several patients with diabetes mellitus. The majority of these patients had insulin‐dependent diabetes, accompanied by severe vomiting, and signs of hypovolemia and tachycardia at initial presentation. Following the administration of insulin and intravenous fluid, clinical symptoms were rapidly improved.^7^, ^8^, ^9^, ^10^

Metabolic alkalosis is a frequent metabolic disturbance encountered in emergency departments. Generally, reduced intravascular volume is the common etiology of this condition, which leads to many general practitioners to address it solely with intravenous normal saline administration. Nonetheless, the absence of an anion gap calculation can result in a delay in diagnosing and effectively managing underlying causes of wide anion gap metabolic acidosis, such as DKA or exposure to toxins. General practitioners should remain vigilant about the possibility of masked wide anion gap metabolic acidosis within the context of metabolic alkalosis.

## 6 CONCLUSION

The approach to patients with metabolic alkalosis should include a detailed history, volume status assessment, and urine electrolyte to guide the etiology of metabolic alkalosis. Understanding these mechanisms that generate and maintain alkalosis may support diagnosis and treatment more precisely. Moreover, even in documented metabolic alkalosis, the anion gap should be regularly calculated to detect masked wide anion gap metabolic acidosis as shown in this case.

## AUTHOR CONTRIBUTIONS

**Thunyatorn Wuttiputhanun:** Conceptualization; investigation; visualization; writing – original draft. **Natavudh Townamchai:** Supervision; writing – review and editing. **Somchai Eiam‐Ong:** Supervision; writing – review and editing. **Kullaya Takkavatakarn:** Conceptualization; supervision; writing – review and editing.

## FUNDING INFORMATION

The authors received no financial support for the research, authorship, and/or publication of this article.

## CONFLICT OF INTEREST STATEMENT

The authors have no conflict of interest to declare.

## CONSENT

Written informed consent was obtained from the patient to publish this report in accordance with the journal's patient consent policy.

## Data Availability

Data and materials may be made available upon written request in the corresponding author.
